# An S1-Nanoparticle Vaccine Protects against SARS-CoV-2 Challenge in K18-hACE2 Mice

**DOI:** 10.1128/jvi.00844-22

**Published:** 2022-06-29

**Authors:** Linda van Oosten, Kexin Yan, Daniel J. Rawle, Thuy T. Le, Jort J. Altenburg, Cyrielle Fougeroux, Louise Goksøyr, Willem Adriaan de Jongh, Morten A. Nielsen, Adam F. Sander, Gorben P. Pijlman, Andreas Suhrbier

**Affiliations:** a Laboratory of Virology, Wageningen University, Wageningen, The Netherlands; b QIMR Berghofer Medical Research Institute, Brisbane, Australia; c Bioprocess Engineering, Wageningen University, Wageningen, The Netherlands; d AdaptVac Aps, Hørsholm, Denmark; e Centre for Medical Parasitology at Department for Immunology and Microbiology, Faculty of Health and Medical Sciences, University of Copenhagen, Copenhagen, Denmark; f GVN Center of Excellence, Australian Infectious Disease Research Centre, Brisbane, Australia; University of North Carolina at Chapel Hill

**Keywords:** SARS-CoV-2, nanoparticle vaccine, K18-hACE2

## INTRODUCTION

**FIG 1:**
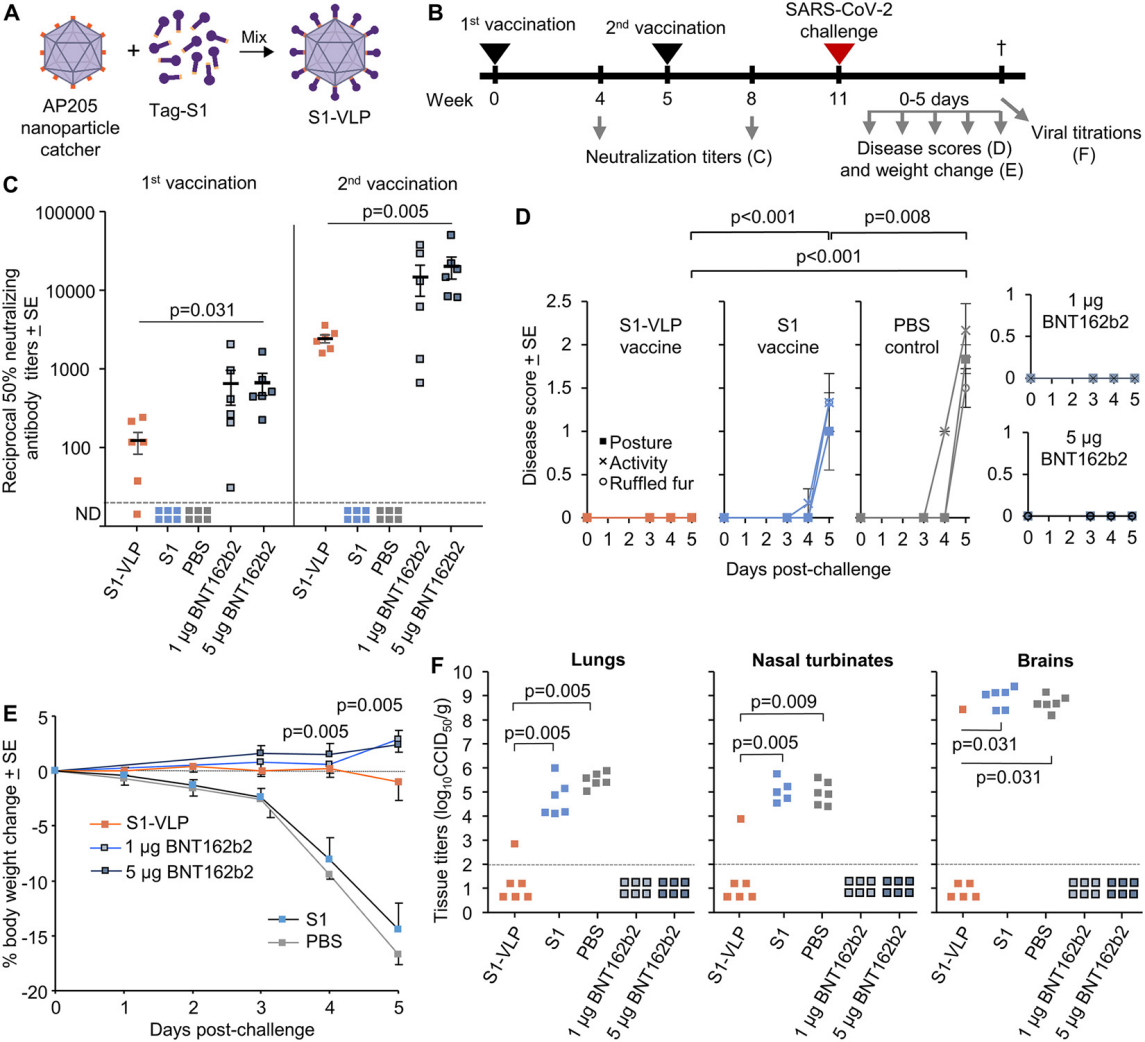
Vaccination and challenge of K18-hACE2 mice. (A) Vaccine-comprised S1 protein coupled onto AP205 nanoparticles by tag-catcher covalent isopeptide bond. (B) Experimental timeline. K18-hACE2 mice (*n* = 6/group) were vaccinated with two doses of 2 μg S1-VLP, 2 μg S1 subunit, 50 μL phosphate-buffered saline (PBS), 1 μg BNT162b2, or 5 μg BNT162b2. Time of challenge, blood collection, clinical disease score monitoring, and viral load determinations are indicated. (C) Serum was collected after each vaccination and neutralizing titers determined. Limit of detection, 1 in 20 dilution of serum. (D) Clinical disease scores were monitored for posture, activity, and fur ruffling (means are shown). (E) Mean percentage weight change per group after challenge relative to the mice’s weight on day 0. *P* values shown for S1-VLP versus S1 or PBS on days 4 and 5. (F) On day 5 postchallenge, SARS-CoV-2 viral titers in lungs, nasal turbinates, and brains were determined. Each dot represents a single mouse in panels C and F. In panels D and E, the average scores per group are shown. Statistics throughout by Kolmogorov-Smirnov tests.

The SARS-CoV-2 pandemic has seen the rapid development and deployment of new vaccines and vaccine technologies. One of the latter involves the use of nanoparticle or virus-like particle (VLP) technologies, with the display of recombinant SARS-CoV-2 spike protein, the S1 domain, or the receptor-binding domain (RBD) on VLPs or nanoparticles providing immunogenic COVID-19 vaccines (1). We previously described a two-component nanoparticle vaccine based on the SARS-CoV-2 spike S1 domain (2). Correctly folded and highly glycosylated S1 (strain Wuhan) was expressed *in* Spodoptera frugiperda (ExpiSf9) insect cells using the well-established baculovirus expression vector system (BEVS), with S1 displayed on bacteriophage AP205 capsid-like nanoparticles via a tag-catcher covalent bond (2) (Fig. 1A). This S1-VLP nanoparticle vaccine was immunogenic at a 0.5-μg dose (formulated with Addavax adjuvant) in BALB/c mice. A related vaccine, ABNCoV2, confers protection in rhesus macaques (3) and is currently in stage III clinical trials (ClinicalTrials.gov identifier NCT05329220) and comprises the RBD displayed on the AP205 nanoparticles (4). The AP205 nanoparticles encapsulate bacterial RNA, which provides adjuvanting activity via engagement of Toll-like receptors 7 and 8 (TLR7/8) (4).

We have now undertaken a vaccination and challenge study in K18-hACE2 transgenic mice (Fig. 1B), which provide a robust and lethal model of SARS-CoV-2 infection (5–7); ethics statements and regulatory compliance and detailed methods are available in Rawle et al. (8). Female K18-hACE2 mice (*n* = 6/group) received two intramuscular vaccinations 5 weeks apart with 2 μg S1 (no adjuvant), 2 μg S1-VLP (no adjuvant), or phosphate-buffered saline (PBS). As positive controls, mice were double vaccinated with 1 μg or 5 μg of the licensed SARS-CoV-2 mRNA vaccine (BNT162b2; Pfizer-BioNTech) (9); we used the discarded remnant in the multi-dose vial after 6 doses had been used to vaccinate humans. Serum-neutralizing antibody (nAb) titers were quantified (8) after the first and second immunizations (Fig. 1C). The S1 subunit vaccine did not induce detectable nAb titers. In contrast, mice vaccinated with S1-VLP and BNT162b2 produced robust nAb titers that increased substantially after the second vaccination (Fig. 1C). The responses induced by S1-VLP vaccination were not significantly different from those induced by 1 μg BNT162b2, but were significantly lower than those induced by 5 μg BNT162b2 (Fig. 1C).

At 11 weeks after the first vaccination (Fig. 1B), the mice received an intrapulmonary challenge delivered intranasally with 5 × 10^4^ 50% cell culture infectious dose (CCID_50_)/mouse (in 50 μL) SARS-CoV-2 UK strain, B1.1.7; hCoV-19/Australia/QLD1517/2021 (10), and disease scores (quantitating overt clinical signs [6]) and weight change were monitored over 5 days. Mice that received the S1 subunit vaccine or PBS developed clear signs of disease (Fig. 1D) and showed significant weight loss (Fig. 1E). Mice vaccinated with the S1-VLP vaccine or BNT162b2 were fully protected from signs of disease (Fig. 1D) and weight loss (Fig. 1E). At 5 days postchallenge, SARS-CoV-2 tissue titers were determined in the lungs, nasal turbinates, and brains (8). Vaccination with S1-VLP and BNT162b2 vaccines significantly reduced the viral loads in these target organs, although one S1-VLP-vaccinated mouse was not fully protected against detectable virus (Fig. 1F).

These data show that our previously described two-component nanoparticle vaccine S1-VLP (2) provides protective immunity in mice against SARS-CoV-2 infection and disease without a requirement for additional adjuvants. In contrast, the S1 subunit vaccine, not conjugated to nanoparticles, provided no significant protection. The S1-VLP vaccine induced neutralizing antibody responses that were comparable with those induced by 1 μg BNT162b2. This VLP display platform should theoretically be readily amendable to any (or even multiple) SARS-CoV-2 variants of concern.
